# Watching TV and Cognition: The SPAH 2-Year Cohort Study of Older Adults Living in Low-Income Communities

**DOI:** 10.3389/fneur.2021.628489

**Published:** 2021-06-25

**Authors:** Laís Fajersztajn, Vanessa Di Rienzo, Carina Akemi Nakamura, Marcia Scazufca

**Affiliations:** ^1^Laboratório de Poluição Ambiental, Departamento de Patologia, Faculdade de Medicina (FMUSP), Universidade de São Paulo, São Paulo, Brazil; ^2^Faculdade de Medicina (FMUSP), Universidade de São Paulo, São Paulo, Brazil; ^3^Universidade São Judas Tadeu, São Paulo, Brazil; ^4^Laboratório de Investigação Medica (LIM) 23, Faculdade de Medicina, Instituto de Psiquiatria, Hospital das Clinicas HCFMUSP, Universidade de São Paulo, São Paulo, Brazil

**Keywords:** dementia, mild cognitive impairment, older adults, leisure activities, low-income, television, cognitive dysfunction

## Abstract

Watching TV is a highly prevalent leisure activity among older adults and, in many cases, the only leisure option of those living in low-income communities. While engaging in leisure activities have proven to protect older adults from cognitive decline, the effects of watching TV on cognition of this population is controversial in the literature. This study investigated the impact of watching TV on global cognitive function, immediate memory, verbal fluency, risk of dementia of amnestic mild cognitive impairment (aMCI) in a cohort of older adults residents of socioeconomically deprived areas of São Paulo, Brazil. We used data from the São Paulo Aging & Health Study (SPAH). Participants aged 65 years or over, with no dementia diagnosis at baseline and who completed the 2-year follow-up assessment were included in this study (*n* = 1,243). Multivariable linear regression models were performed to assess the effect of watching TV on global cognitive function, immediate memory and verbal fluency. Multivariable logistic regression models were used to evaluate the risk of developing dementia and aMCI. Models were controlled by cognitive performance at baseline, sociodemographic characteristics and functional status. Cognitive performance at baseline and follow-up were similar. Thirty-one participants were diagnosed with dementia, and 23 with aMCI 24 months after inclusion in the study. Watching TV did not show any positive or negative effect on global cognitive function, immediate memory, verbal fluency, risk of dementia and risk of aMCI. It is good news that watching TV did not predict the decline in cognition in elders. However, it is essential to increase opportunities for other leisure activities for low-income and low-educated older adults if we do consider that leisure activities protect cognition decline in older adults. In the coming decades, developing countries will experience the highest burden of dementia and more than fun, public policies to promote leisure activities might be a strategy to alleviate this burden shortly.

## Introduction

More than 28 million Brazilians -or 13% of the country's population- are aged 60+, and the size of this age group is projected to double in the coming decades (1). The Brazilian elderly population has been exposed to socioeconomic adversities throughout their life course (2, 3). Most of the Brazilian elders have minimum or no formal education, had semi-skilled or non-skilled occupations throughout their lives, are poorer than the average adult population of the country and were raised in rural areas before moving to large cities during the second half of the 20th century (4). It means that they are at a higher risk of developing cognitive related health problems (2, 5). Currently, as many as 6 million Brazilians older adults cannot read or write (6). These characteristics influence the type of leisure activity performed by them.

By 2050, Latin America will host more than 17 million dementia cases (7), and strategies to reduce the burden of cognitive impairment in the region are badly needed. One possible response is to reduce social inequalities by improving modifiable risk factors for cognitive decline, such as education, quality of occupation and socioeconomic status. Social, physical and intellectual leisure activities have shown to reduce the risk of cognitive impairment (8–14) and dementia in older adults (9, 10, 15, 16), but the ability to perform these activities is affected by socioeconomic difficulties faced during the life course (17–19).

Although watching TV is an everyday leisure activity among older adults (20) worldwide, the effects of this activity on their cognition are controversial in the literature. There is no consensus in the literature of to which extent watching TV is a cognitively demanding activity or what type of activity is watching TV (e.g., is it a recreational activity?) (21–23). Longitudinal studies in Europe and the US suggest that excessive hours watching TV impairs cognition somehow (24, 25). Some studies didn't see a longitudinal effect (26, 27). However, one large study in Asia found a protective effect (11).

To date, little (28) is known of the potential protective effects of leisure activities among Latin American populations, particularly from low-income communities, where leisure activities significantly differ from those performed by older adults in high income countries. The low purchase power, restricted mobility and low literacy of this population are factors that restrict their leisure options. In this context, watching TV becomes an essential and almost the only leisure activity available to most Brazilians older adults. In Brazil, watching TV is the most frequent leisure option of 93% of the adults aged 60+ (29).

Given the importance of watching TV as a leisure activity among older adults from low-income communities and the lack of empirical data on its effect on cognition in these population, we examined the association between watching TV and incidence of amnesic mild cognitive impairment (aMCI) and dementia in a 2-year cohort study of older adults residents of socioeconomically deprived areas of São Paulo, Brazil. We also examined the association between watching TV and changes in global cognitive function, immediate memory, verbal fluency in the same population.

## Materials and Methods

This study is part of the São Paulo Aging & Health Study (SPAH), a large 2-year population-based cohort study of older adults from low-income areas of São Paulo, Brazil (2, 30).

### Participants

SPAH enrolled 2,072 community-dwellings aged 65+ that lived in socioeconomically deprived areas of the district of Butantã, in São Paulo, Brazil. The recruiting method was knocking on the door of all households within the 66 pre-defined census sectors (the smallest administrative areas) between 2003 and 2005. The census sectors selected a priori were those with the lowest Human Development Index, including large informal settlements and shantytowns. Follow-up occurred 24 months after enrollment, between 2005 and 2007. Trained interviewers conducted the baseline and follow-up assessments at participants' home, using the SPAH protocol. More details of recruitment procedures are described elsewhere (2, 3, 30).

Participants without dementia at baseline and who could complete the follow-up assessment were included in this study. Socioeconomic and demographic information (age, gender, marital status, schooling, occupation, and personal income) of all participants were collected at baseline assessment. We used the 12-item World Health Organization Disability Assessment Schedule (WHODAS 2.0) to assess functional status (31). The WHODAS 2.0 is a questionnaire that evaluates six areas of life: mobility (moving and getting around), life activities (domestic responsibilities, leisure, work, and school), cognition (understanding and communicating), self-care (hygiene, dressing, eating, and staying alone), participation (joining in community activities), and getting along (interacting with other people). The score ranges from 0 to 48, and the higher the score, the more severe is the disability.

The study received ethics approval from the Brazilian National Committee for Ethics and Research (CONEP-Brazil), and all participants provided written informed consent before enrollment in the study.

### Outcomes

The five clinical outcomes in this study are global cognitive function, immediate memory, verbal fluency, risk of dementia and risk of amnestic mild cognitive impairment (aMCI).

#### Global Cognitive Function

We used thecognitive score (COGSCORE) validated by the 10/66 Dementia Research Group to evaluate global cognitive function. The score is based on the Community Screening Instrument for Dementia (CSD-I) (32), an instrument developed for low educated and illiterate populations (33) and validated for use in Brazil (34). The global cognitive function includes memory, abstract thinking, language, praxis, space and temporal orientation dimensions. Higher scores indicate more severe impairments.

#### Immediate Memory

We evaluate immediate memory by asking participants to memorize a list of 10 words adapted from the Consortium to Establish a Registry for Alzheimer's Disease (CERAD) battery (35). The total score is based on the number of recalled words, thus maximum score possible is 10 representing the best immediate memory.

#### Verbal Fluency

We used animal naming to evaluate verbal fluency. This test is part of the CERAD battery (35) and the CSI-D (32). First, we asked the participant to name items from another category (dressing). After this test, the participant was asked to name all animals he/she could remember during 1 min. Each animal named equals one point. Higher scores indicate better performance on the task.

#### Dementia

The diagnosis of dementia followed the Diagnostic and Statistical Manual of Mental Disorders, 4th Edition (DSM-IV) (36). It was based on the protocol developed by 10/66 Dementia Research Group for population-based studies in developing countries (34). Detailed description can be found elsewhere (2, 30).

#### Amnestic Mild Cognitive Impairment

This study used two conditions to determine the Amnestic Mild Cognitive Impairment diagnosis (aMCI): absence of dementia and memory impairment (37). We considered an individual with memory impairment if his/her total score in the memory test was at least 1.5 standard deviation (SD) below the study population's mean. The memory test score was based on four items of the cognitive dimension of the Community Screening Instrument for Dementia (CSD-I) (32). Participants were required to recall the interviewer's name, repeat three standardized words, recall these words and repeat stories. Scores are positively associated with memory performance.

### Predictor Variable

The predictor variable was time spent watching TV assessed by asking “How many hours per day you watch TV?” We collected this information at baseline using the Brazilian version of the 'Involvement in Activities' questionnaire (IA). The IA was developed to evaluate engagement in activities in diverse communities and was used in the United States and Nigeria (38). The Brazilian version of the IA was validated in Brazil by the SPAH group in collaboration with the authors who developed the questionnaire. The test-retest reliability of the IA included 70 participants. They were re-assessed with an interval of 3–4 weeks. The reliability of the question about hours spent watching TV was high (0.63).

### Statistical Analysis

We used multivariable linear regression to examine the association between watching TV at baseline and global cognitive function, immediate memory and verbal fluency at 2-year follow-up. Two models were built for each outcome. In the first models, we entered hours watching TV adjusting for the corresponding baseline score. Then, we extended these models by adjusting for sociodemographic characteristics (age, gender, marital status, schooling, occupation, and personal income) and function status (second models).

To investigate the impact of watching TV on the risk of dementia and aMCI, we performed two multiple logistic regression models for each outcome variable. We adjusted the first models for sociodemographic characteristics and functional status at baseline. We then extended the models for risk of dementia and the model for aMCI adjusting for global cognitive function at baseline amnestic aMCI at baseline, respectively. We present the 95% confidence interval (CI) for all analysis and set statistical significance at 5%.

Statistical significance was set at 5% for all analysis. We performed the analyses with the software STATA 9.0.

## Results

A total of 1,243 subjects without the dementia diagnosis at baseline who completed the 2-year follow-up assessment were included in this study. Participants were most females (61%), with a mean age of 72 years, mainly not employed (73%), low educated (89% with up to 3 years of schooling) and with a low socioeconomic background (only less than one third had a personal monthly income of more than US$246). Participants' characteristics at baseline are presented in Table 1. The median functional status score was 2.8 (IQR = 0–72.2). Watching TV was reported by 87% of participants with a median of 2 h per day (IQR = 0–12). Thirteen percent of the participants reported never watching TV (*n* = 165). Among those whom reported watching TV, 22.2% watched up to an hour/day (*n* = 276); 22.7% watched up to 2 h/day (*n* = 282); 14.9% watched up to 3 h/day (*n* = 185); 11.3% watched up to 4 h/day (*n* = 141); 7.4% watched up to 5 h/day (*n* = 92); and 8.2% watched 6 h/day or more (*n* = 102).

**Table 1:** Sociodemographic and functional characteristics of the participants of the study (*n* = 1,243).

**Baseline characteristics**	
Age (years), mean ± SD^*^	71.7 ± 5.8
Gender (female), *n* (%)	763 (61.4)
**Schooling (years)**, ***n*** **(%)**	
None	401 (32.3)
1–3	701 (56.4)
4 and over	141 (11.3)
Marital status (married), *n* (%)	574 (46.2)
**Occupation (employment)**, ***n*** **(%)**	
None	910 (73.2)
Partial time	221 (17.8)
Full time	112 (9.0)
**Personal income**, ***n*** **(%)**	
up to 1 MW^*^^a^	276 (22.2)
>1–1.5 MW^*^^a^	251 (20.2)
>1.5–3 MW^*^^a^	346 (27.8)
> 3 MW^*^	370 (29.8)
Functional status (WHODASII), median (IQR)	2.8 (0–72.2)
Watching TV, median (IQR)	2 (0–12)

* *IQR, interquartile range; MW, minimum wage; SD, standard deviation.*

a *At the time of the study, the Brazilian monthly minimum wage was US$85*.

The cognitive performance of participants was similar between baseline and follow-up assessments (Table 2). At follow-up, mean global cognition function, immediate memory, and verbal fluency scores were 27.1 ± 4.3, 3.2 ± 1.3, and 12.8 ± 4.5. Watching TV showed no association with any cognitive outcome when controlling only by the cognitive performance at baseline or in combination with sociodemographic characteristics and functional status (Table 3).

**Table 2:** Mean and standard deviation of the cognitive performance of the participants at baseline and follow-up (*n* = 1,243).

	**Baseline**	**Follow-up**
Global cognitive function (CSI-D)	27.7 ± 3.4	27.1 ± 4.3
Immediate memory (10 words list–CERAD)	3.0 ± 1.3	3.2 ± 1.3
Verbal fluency (animal naming–CERAD)	12.9 ± 4.3	12.8 ± 4.5

*CERAD, Consortium to Establish a Registry for Alzheimer's Disease; CSI-D, Community Screening Instrument for Dementia*.

**Table 3:** Multivariable regression models of cognition performance at follow-up on watching TV (hours/day) adjusting by the corresponding cognition metric at baseline (model 1); and adding sociodemographic characteristics and functional status at baseline (model 2).

	**Model 1 (*****n*** **=** **1,243)**		**Model 2 (*****n*** **=** **1,243)**	
	**Coefficient** (95% CI^*^)	***p*-value**	**Coefficient** (95% CI^*^)	***p*-value**
Global cognitive function	0.30 (−2.90, 3.50)	0.90	0.60 (−2.60, 3.80)	0.70
Immediate memory	0.02 (−0.01, 0.05)	0.20	0.01 (−0.02, 0.05)	0.40
Verbal fluency	−0.02 (−0.10, 0.07)	0.60	−0.02 (−0.10, 0.07)	0.70

* *CI, confidence interval*.

Thirty-one participants were diagnosed with dementia during the follow-up assessment. No association between watching TV and the risk of developing dementia in 2 years was observed in the two models. Amnestic—was identified in 23 participants 2 years after the baseline assessment, and watching TV also showed no effect on preventing or increasing the risk of developing this condition (Table 4).

**Table 4:** Logistic regression models of the risk of dementia and amnestic mild cognitive impairment on watching TV (hours/day), sociodemographic characteristics and functional status at baseline (model 1); and adding baseline global cognitive function for dementia relative risk and the corresponding baseline score for amnestic mild cognitive impairment (model 2).

	**Model 1 (*****n*** **=** **1,243)**		**Model 2 (*****n*** **=** **1,243)**	
	OR^*^ (95% CI^*^)	***p*-value**	OR^*^ (95% CI^*^)	***p*-value**
Dementia relative risk	0.81 (0.64, 1.02)	0.05	0.82 (0.63, 1.04)	0.08
Amnestic mild cognitive impairment	1.00 (0.82, 1.21)	0.97	1.02 (0.84, 1.24)	0.80

* *CI, confidence interval; OR, odds ratio*.

## Discussion

In our study, hours per day watching TV did not impact positively or negatively the global cognitive function, immediate memory or verbal fluency of older adults after 2 years. Watching TV did not increase the risk or protect participants of developing dementia or aMCI after 2 years of the initial assessment.

Despite being such a common leisure activity among older adults (20)—and frequently the only leisure option for low-income and low-educated older adults-, not many studies investigated the isolated effect of watching TV on cognition (11, 24, 25, 27, 39) and the findings are controversial.

Our results are in accordance with an English (27) and a French study (26). In the English cohort study (*n* = 6,359 older adults aged 65 or over, 2-year follow-up) (27), more time watching TV was associated with poorer global cognition at baseline, but not with changes after 2 years. There is no specific information about the educational and socioeconomic status of the population in the English cohort. Our study also enrolled 65+ individuals for the same follow-up period. The Frenchstudy (26) enrolled 2,579 adults aged between 45 and 60 years old and found that more time watching TV was associated with worse executive function at baseline, but not with verbal memory. All participants in the French study were over 60 years old and no changes were observed after 6 years. Compared to the French study, ours enrolled adults with higher ages (65+ vs. 60+ years old) but a shorter follow-up (2 vs. 6 years).

Different from our results, three studies found that excessive hours watching TV impairs at least one domain of cognition (24, 25, 39). One of the studies is a large English cohort (*n* = 3,662, 6 years of follow-up) (24), where more hours watching TV was associated with a decline in verbal memory but not in semantic fluency. A possible reason why our results differ from theirs are differences in the population studied. While the English study enrolled adults aged 50+, we enrolled adults aged 65+. Unlike the English study, ours enrolled low-income older adults for whom watching TV is the main leisure activity (more likely exposed). The elders in our study also have less formal education, resulting in fewer opportunities to be exposed to highly cognitive stimulus that would compensate the passiveness of watching TV. The follow-up period in the English study was three times larger. The other two studies that found an association between watching TV and cognitive impairment are less robust. One is a European cross-sectional study (39), and the other is a case-control study (135 cases and 331 controls) (25) that investigated the incidence of Alzheimer Disease in the US.

One study, however, found a protective effect (11). The result of this study differs from the others in the literature and ours. It is a large Chinese cohort (*n* = 6,586, 6 years follow-up), where watching TV was associated with a lower risk of cognitive decline (11). These authors argue that the difference in their results might be because their population is low educated (<6 years of education), and watching TV could act as a cognitive stimulus for them. If this is true, we would expect that watching TV would protect the brain of our population, given that 89% of the population of our study have up to 3 years of schooling.

An open question in the literature is if watching TV in later life could be a cognitive stimulus. If this is true, more time watching TV would build resilience and protect the brain. However, this is not what we observed in our cohort of low-income elders exposed to watching TV as their main leisure activity in adult life. It is likely that the type of program watched, not measured by our study, plays a vital role in the effect of watching TV on cognition. For example, watching a soap opera result in different a more passive stimulus for the brain, compared to watching an educational documentary on a novel subject (40). Indeed, preferring to watch soap operas and talk shows over documentaries, news, sports and other programs was associated with worse performance in cognitive tests in a cross-sectional study (40). Not accounting for the type of program watched is a limitation of our study. However, all longitudinal studies investigating the impacts of watching TV on the cognition of older adults have the same limitation (21, 24, 26, 27). They focus on the frequency of watching TV and do not account for the type of TV program. The only study that accounted for the kind of TV program is a smaller cross-sectional study (40). Given the low-income and low educational level of the population in our study, we can speculate that the programs watched were more similar to the soap opera than the documentary. By the time of the research, cable TV and the internet were not widely available.

Our study is the first study to evaluate the impacts of watching TV on the cognition of older adults living in socioeconomically deprived areas of South America. Although our study has a shorter follow-up period (2 years) compared to most of the prospective studies on the topic literature [ranging from 2 to 9 years follow-up (24, 26, 27, 41)], it is the only prospective study investigating the incidence of dementia.

The average Brazilian older adult is poor, low educated and with reduced leisure options compared to elder populations in developed countries, where most of the studies on healthy aging take place. In developed countries, the most frequent leisure activities among older adults seem to be reading, enrolling in courses, visiting museums, theater, or movies (42–44). In Brazil, the most frequent leisure activities are less complex: watching TV and listening to the radio (29). These activities are not likely to have the same protective effect on cognition as more complex activities might have. Another important reason for not engaging in other leisure activities is purchase power. Mobility might play a role given 35% of Brazilians aged 60+ reported difficulties walking in streets, and 4% reported never living the house (29).

Watching TV is a highly popular leisure activity among the population studied and among other older adults living in socially deprived urban areas of Latin America and other underserved regions. For most of these older adults watching TV is the only leisure option. The good news supported by our data is that watching TV did not predict a decline in cognition, as pointed out by a Chinese cohort (11). Thus, our data do not support advocacy against watching TV among low-income and low-educated older adults. However, in our study, watching TV did not protect from cognitive decline either.

The literature shows that leisure activities can prevent cognitive decline in older adults (16, 45) and should be encouraged through public policies. Thus, it is imperative to increase other leisure activities beyond watching TV for low-income and low-educated older adults. Leisure activities that they can engage in and benefit from.

Performing different types of activities, rather than always the same activity, seems to play a role in preserving cognitive function (46), another strong point to support the promotion of leisure activities for older adults of socially deprived areas. In the coming decades, developing countries will experience the highest burden of dementia and more than entertainment, public policies to promote leisure activities might be a strategy to alleviate this burden shortly.

## Data Availability Statement

The raw data supporting the conclusions of this article will be made available by the authors, without undue reservation.

## Ethics Statement

The studies involving human participants were reviewed and approved by Brazilian National Committee for Ethics and Research (CONEP-Brazil). The patients/participants provided their written informed consent to participate in this study.

## Author Contributions

VD and MS designed the study, supervised data collection, planned and carried out the statistical analyses, and reviewed drafts the paper. MS drafted the paper. LF and CN contributed to the interpretation of the results and drafting of the paper. All authors approved the final version of the manuscript and agreed to be accountable for the content of the work.

## Conflict of Interest

The authors declare that the research was conducted in the absence of any commercial or financial relationships that could be construed as a potential conflict of interest.
